# Adrenocortical Production Is Associated with Higher Levels of Luteinizing Hormone in Nonobese Women with Polycystic Ovary Syndrome

**DOI:** 10.1155/2014/620605

**Published:** 2014-05-07

**Authors:** Luciana Tock, Gláucia Carneiro, Andrea Z. Pereira, Sérgio Tufik, Maria Teresa Zanella

**Affiliations:** ^1^Division of Endocrinology, Department of Medicine, Universidade Federal de São Paulo, São Paulo, SP, Brazil; ^2^Department of Psychobiology, Sleep Disorders Center, Universidade Federal de São Paulo, São Paulo, SP, Brazil

## Abstract

*Objective.* Insulin resistance (IR) and ovarian and adrenal hyperandrogenism are a common finding in women with polycystic ovary syndrome (PCOS). The aim of the present study was to access possible differences in insulin resistance, gonadotropins, and androgens production in obese and nonobese PCOS women. * Study Design.* We studied 37 PCOS women (16 nonobese and 21 obese) and 18 nonobese controls. Fasting glucose, insulin, androgens, and gonadotropins levels were determined. Salivary cortisol was measured basal and in the morning after dexamethasone (DEX) 0.25 mg. * Results.* Nonobese PCOS women showed higher basal salivary cortisol and serum dehydroepiandrosterone sulfate and luteinizing hormone (LH) levels than controls and obese PCOS. These hormones levels did not differ between the obese and control groups. After DEX administration no differences were found between the three groups. In PCOS women, salivary cortisol levels showed negative correlation with BMI (*r* = −0.52; *P* = 0.001) and insulin (*r* = −0.47; *P* = 0.003) and positive correlation with LH (*r* = 0.40; *P* = 0.016). *Conclusion.* Our results show an increased adrenocortical production in nonobese PCOS women, not related to IR and associated with a normal hypothalamic-pituitary-adrenal suppression. Higher LH levels might be involved in this event.

## 1. Introduction

Ovarian hyperandrogenism is a hallmark of polycystic ovary syndrome (PCOS) and insulin resistance (IR) is a common finding in these women [1–3]. IR is present in 50 to 70% of PCOS women and plays a significant role in the physiopathology of this syndrome. The resultant increases in insulin levels stimulate ovarian androgen production, acting directly on ovarian theca cells, and also reduces sex hormone-binding globulin (SHBG) which increases free androgens levels [1–3]. Furthermore, hyperinsulinism decreases hepatic production of insulin-like growth factor-binding proteins (IGFBPs), leading to increase in insulin-like growth factor 1 (IGF-1) bioavailability which also stimulates ovarian androgen production [1–3]. Insulin also can bind IGF-1 receptor and activate the intracellular events contributing to the ovarian steroidogenic abnormalities found in PCOS women [1–3].

Functional adrenal hyperandrogenism is found in approximately half of PCOS women [1]. These women demonstrate a generalized hypersecretion of adrenocortical products, both in a basal condition and in response to adrenocorticotropic hormone (ACTH) stimulation [4–8], although the mechanisms of these abnormalities are still unclear. Similarly unclear are the effects of insulin resistance on adrenal function in PCOS women [1–9].

Although the prevalence of insulin resistance in women with PCOS is higher compared to healthy women with similar body mass index (BMI), there is a distinguished number of PCOS women, mainly the less obese, that do not show insulin resistance [3]. Presumably a different physiopathology is present in these women.

The aim of the present study was to access possible differences in insulin resistance, gonadotropins, and androgens production in obese and nonobese PCOS women and the influence of insulin resistance on the hormonal profile of these two groups of women. A low-dose (DEX) (0.25 mg) suppression test with measurements of salivary cortisol, which closely correlates with the concentration of serum free cortisol [10], was used to evaluate the hypothalamic-pituitary-adrenal (HPA) axis. This test induces a more modest ACTH suppression than the standard DEX (1.0 mg) test, enabling the detection of subtle differences in HPA axis feedback sensitivity to glucocorticoids [11].

## 2. Materials and Methods

### 2.1. Population

Our study included 37 PCOS women who were divided in 2 groups: 16 nonobese (BMI <30 Kg/m²) and 21 obese (BMI ≥30 Kg/m²) and 18 nonobese healthy women with age between 16 and 45 years. Subjects were recruited from the Endocrinology Division of Federal University of São Paulo (UNIFESP), Brazil. The diagnosis of PCOS was based on the latest 2003 Rotterdam consensus [12] requiring the presence of at least two of the following features: (1) oligomenorrhea or chronic anovulation, (2) clinical and/or biochemical hyperandrogenism, and (3) ultrasound (US) appearance of polycystic ovaries, after the exclusion of other known causes of hyperandrogenemia such as congenital adrenal hyperplasia, androgen-secreting tumors, and Cushing's syndrome. Exclusion criteria included the use of oral contraceptives, corticosteroids, and antidiabetic drugs in the last 3 months. We also excluded patients with diabetes mellitus, untreated hypothyroidism, and renal, hepatic, cardiac, or pulmonary disease. The study was approved by the Ethics Committee of UNIFESP, and an informed written consent was obtained from all subjects.

### 2.2. Clinical, Anthropometrical, and Biochemical Parameters

A questionnaire was used to document personal, medical, and drug history, regularity and length of menstrual cycles, and ovulation status. Signs of androgen excess (hirsutism, alopecia, and acne) were noted in the physical examination. Hirsutism with a Ferriman-Gallwey score of 8 or above was considered as clinical evidence of androgen excess. Body weight (in kilograms), body height (in meters), and waist and hip circumference (in centimeters) were measured. Waist circumference was taken as the narrowest measurement midway between the top of the iliac crest and the lower rib margin, whereas the hip circumference was taken as the widest measurement at the level of the greater trochanters. BMI was calculated from the ratio between weight and height squared.

Blood specimens were obtained after a 12-hour overnight fast from all subjects in the early follicular phase of the menstrual cycle or after a period of amenorrhea above 3 months for measurement of plasma glucose, luteinizing hormone (LH), follicle-stimulating hormone (FSH), androgenic profile, and serum insulin. A standard 75-gram oral glucose tolerance test (OGTT) was performed in PCOS patients for insulin sensitive index (ISI) calculation.

The assessment of HPA axis included low-dose (0.25 mg) DEX suppression test with measurement of salivary cortisol. The women received two Salivettes (Sarstedt, Rommelsdorf, Germany) which consist of a small cotton swab inside a centrifugation tube used to collect saliva and a half tablet of 0.5 mg of dexamethasone (Decadron, Aché, Brazil). Salivary sample was obtained between 7:00 and 8:00 am in the day of the administration of DEX, which was taken between 11:00 and 12:00 pm. Another salivary sample was obtained on the next morning between 7:00 and 8:00 am. To analyze the results we used an index of percentage of salivary suppression (% cortisol suppression) calculated as the difference between the post-DEX cortisol level and baseline cortisol levels divided by baseline cortisol levels and multiplied by 100.

### 2.3. Laboratory Analysis

Plasma glucose was measured using ADVIA 2400 Chemistry System (Siemens, Tarrytown, NY). Dehydroepiandrosterone (DHEA) and 17OH-progesterone levels were measured by enzyme-linked immunosorbent assays (Diagnostic Biochem Canada, Ontario, Canada and Labor Diagnostika Nod GmbH, Nortdhorn, Germany, resp.). Total testosterone (TT), dehydroepiandrosterone sulfate (DHEA-S), LH, FSH, and insulin were measured using UniCel Dxl 800 Immunoassay System (Beckman Coulter, Brea, CA). The within-assay coefficient of variation of testosterone was 1.99%, and the between-assay coefficient was 4.22%. There are some limitations to measuring testosterone using a chemiluminescence immunoassay, but this was the only laboratory technique available. Androstenedione and sex hormone-binding globulin (SHBG) were measured using IMMULITE 2000 Immunoassay System (Siemens). Serum-free testosterone (FT) and bioavailable testosterone were estimated by the formula as previously validated by Vermeulen et al. [13] (available at http://www.issam.ch/freetesto.htm). Free androgen index (FAI) was calculated according to the formula 100 × [TT (nmol/L)/SHBG]. IR was estimated using the homeostasis model assessment for insulin resistance (HOMA-IR) by dividing the product of fasting insulin (microinternational units (*μ*IU)/mL) and glucose (mmol/L) by 22.5. ISI was calculated according to the formula developed by Belfiore et al. [14]: 2/( (INSp × GLYp) + 1), where INSp and GLYp are obtained by dividing the sum of plasma insulin (*μ*IU/mL) and glycemia (mmol/L) (measured at 0 and 2 hours after oral glucose load) by the sum of the respective values of the normal population. Normal reference values were obtained in 35 normotensive individuals with normal BMI. All biochemical assays were performed at the Sleep Institute Laboratory. Salivary cortisol was measured by radioimmunoassay in a single assay run in the Steroid Laboratory of the Endocrinology Division of UNIFESP. The lowest level of detection of salivary cortisol was 5 ng/dL and the between-assay coefficient of variation was 13.1%.

### 2.4. Statistical Analysis

Data are expressed as mean (SD). Comparison of mean values between controls, nonobese, and obese PCOS women was performed using ANOVA. Correlation between variables was determined using linear correlation test. The Fisher test was used to analyze the association between the presence of amenorrhea in the two groups of PCOS. Linear regression analysis was used to identify the hormones associated with cortisol levels among PCOS subjects. A *P* value <0.05 was considered significant. Statistical analyses were performed with the Statistical Package for Social Sciences for Windows, version 19.0 (SPSS Inc, Chicago, IL).

## 3. Results

The anthropometrical and biochemical characteristics of all subjects are summarized in Table 1. The proportions of patients with amenorrhea were not different between the two PCOS groups of patients (25% in nonobese and 33% in obese; *P* = 0.580). Obese PCOS women had higher BMI and waist-to-hip ratio than nonobese PCOS subjects and controls, but no differences were found between controls and nonobese PCOS women.

**Figure 1:**
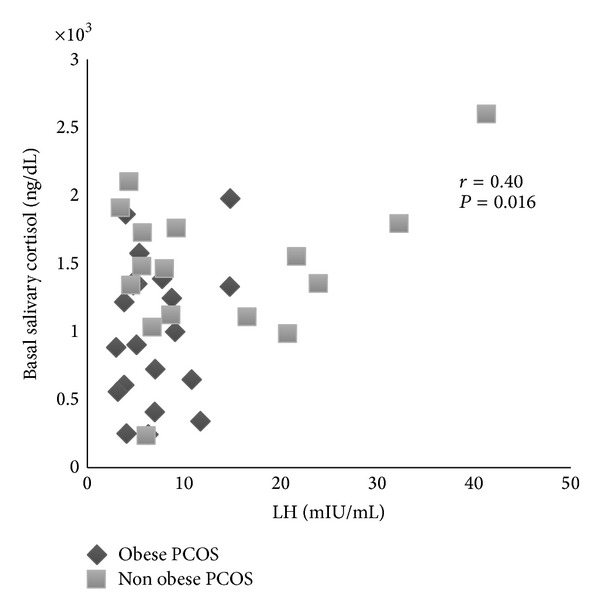
Correlation between basal salivary cortisol and luteinizing hormone (LH) levels in the two groups of women with polycystic ovary syndrome.

Nonobese PCOS women had higher LH, DHEA-S, and basal salivary cortisol (Figure 1) than control and obese PCOS women. These hormones levels were similar between control and obese PCOS women. Although there was a trend for a difference in DHEA levels between the two PCOS groups, this difference did not reach statistical significance (*P* = 0.072).

With regard to androgens profile, total and free testosterones and FAI were similar between PCOS groups and higher than control group although bioavailable testosterone showed to be higher only when obese PCOS group was compared to control with no difference between the two groups of PCOS. Androstenedione was higher in nonobese PCOS women when compared to controls with no difference between the two groups of PCOS.

There were no differences in fasting glucose levels among the three groups although HOMA-IR and fasting insulin levels were higher in obese PCOS group compared to nonobese PCOS and control. There were no statistical differences in salivary cortisol levels after DEX suppression test among the three groups.

**Figure 2:**
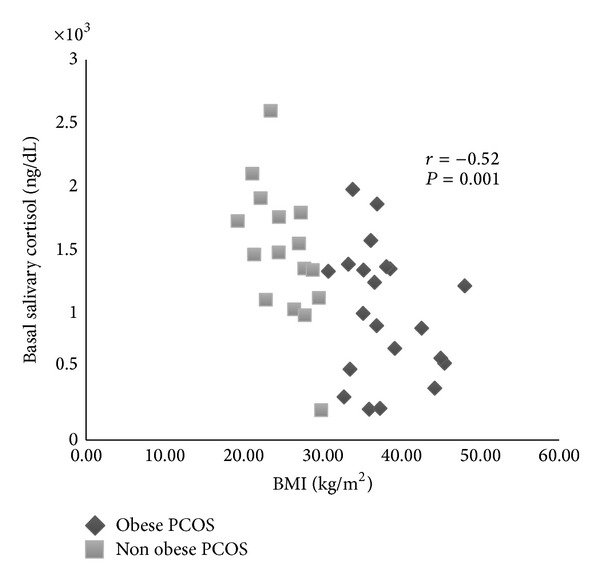
Correlation between basal salivary cortisol levels and body mass index (BMI) in women with polycystic ovary syndrome.

In the total group of PCOS, salivary cortisol levels showed positive and significant correlation with LH levels (*r* = 0.40; *P* = 0.016) (Figure 1) and negative correlation with HOMA-IR (*r* = −0.48; *P* = 0.003), fasting insulin levels (*r* = −0.47; *P* = 0.003), and BMI (*r* = −0.52; *P* = 0.001) (Figure 2). There was a marginally significant positive correlation between basal salivary cortisol and DHEA levels (*r* = 0.31; *P* = 0.061) and DHEA-S (*r* = 0.31; *P* = 0.063) levels. LH levels also showed negative and significant correlation with BMI (*r* = −0.36; *P* = 0.030).

In linear regression analysis among PCOS subjects, with LH and fasting insulin as independent variables and salivary cortisol as dependent variable, both hormones showed to have significant and independent association with cortisol levels: LH showed positive association and insulin negative association (Table 2).

## 4. Discussion

In the present study we showed that there is no hyperactivity of HPA axis in PCOS women as the suppression of salivary cortisol after a low dose of DEX was similar in these women and in controls. The presence of obesity also did not interfere in low-dose DEX suppression.

Studies about cortisol production and metabolism in PCOS subjects demonstrate heterogeneous results. Some of them show normal basal cortisol levels [15–19], while others show higher cortisol plasma levels [5–7, 20], higher ACTH levels [5, 7], and hyperresponsiveness of adrenal to ACTH [4, 7, 8, 17]. A possible explanation for this heterogeneity is the wide range of BMI included in these analyzes. We found higher levels of basal salivary cortisol in nonobese PCOS women compared with obese PCOS and nonobese controls. Our results are partly in accordance with a recent study where lean PCOS had higher basal cortisol levels compared to obese PCOS and lean controls, although LH levels did not show similar pattern [21].

Basal levels of DHEA-S also showed similar pattern for basal levels of salivary cortisol with higher values in nonobese PCOS women compared to obese PCOS and nonobese controls. The other androgens levels were similar in both PCOS groups, and, as expected, they were higher in PCOS women than in controls. DHEA-S is primarily secreted by the adrenals as there is maintenance of its levels in women with premature ovarian failure [22, 23] and absence of this hormone in the ovarian vein after catheterization [20]. These results suggest that adrenals cortex has an important role in androgen production only in nonobese PCOS women.

Another substantial difference between PCOS groups in our study was related to LH levels. Gonadotropin-secretory changes, with a characteristic increase in LH relative to FSH release, have long been appreciated in PCOS. The increase in LH pulse frequency reflects an increase in GnRH release and suggests the presence of a hypothalamic defect in this group of women [1]. We found that nonobese PCOS women had higher levels of LH compared to the other two groups, and the level of this hormone was similar in controls and obese PCOS women. We also found a negative correlation between LH levels and BMI in PCOS women. Based on these results and in previous studies [22, 24–26], we can assume that there are two distinct groups of PCOS: one composed of nonobese, noninsulin resistant women, showing higher levels of LH and adrenocortical hormones (cortisol and DHEA-S) and another composed of obese, insulin resistant women showing lower levels of LH and adrenocortical products. Our control group was composed only of nonobese women, who showed levels of cortisol and LH comparable to those of obese PCOS women.

We found a significant and positive correlation between salivary cortisol and LH levels in PCOS women. This correlation was previously found in postmenopausal women who also present high levels of LH [27, 28]. Several studies have demonstrated the presence of LH receptors in adrenocortical cells which also bind human chorionic gonadotropin (hCG) and their potential to induce local steroidogenesis [29–36]. In situ hybridization and immunocytochemistry also demonstrated that the adrenal zona reticularis and the deeper layer of adrenal zona fasciculada contain LH/hCG receptors [31]. In addition, it has been demonstrated that administration of hCG stimulates cortisol production in guinea-pig adrenal cells [30], increases DHEA-S production in human fetal adrenal gland [29], and stimulates DHEA-S production in human adrenocortical carcinoma cells [35]. Study including 2 women with Cushing's syndrome and bilateral adrenal hyperplasia demonstrated pronounced cortisol rise after GnRH administration and, in vitro, the adrenal cells of these women also responded to hCG exposure increasing cortisol production. Furthermore, LH receptor mRNA was demonstrated in adrenal tissue of both patients [34]. Lacroix et al. [32] reported a woman with bilateral adrenal hyperplasia who presented high cortisol levels and Cushing's syndrome during her pregnancies and permanent hypercortisolism only after menopause. This patient showed increased cortisol secretion in response to LH and hCG administration. In addition, long-term suppression of LH by leuprolide acetate administration resulted in complete reversal of Cushing's syndrome. It raises the possibility of an overexpression of LH receptors in adrenal gland also in nonobese women with PCOS.

Supporting the idea that high LH levels are responsible for the adrenocortical hyperactivity in nonobese PCOS women, Kero et al. [33] showed the development of polycystic ovaries and bilateral adrenal hyperplasia, with increased androgen and cortisol production, in a transgenic mouse model with a high constitutive LH production. Mazzuco et al. [36] demonstrated in a mouse model transplanted with genetically modified bovine adrenocortical cells with an overexpression of LH/hCG receptor gene that it was able to provoke subclinical or mild hypercortisolism.

The finding in our study that nonobese PCOS patients have increased adrenocortical production with normal suppressible cortisol production after low dose of DEX, and a demonstration of a possible influence of LH in adrenal steroidogenesis as described before suggests that LH might influence adrenocortical production in nonobese PCOS women, presumably increasing the adrenocortical response to ACTH. This hypothesis was proposed in 1999 by Lacroix et al. [32] who showed increases in cortisol levels after LH administration, which were proportional to ACTH levels in two healthy women in whom the secretion of endogenous LH had been previously suppressed. The same group demonstrated that the suppression of ACTH by pretreatment with DEX prevented the increase of cortisol after LH injection.

Our group of obese PCOS was more insulin resistant than the nonobese group and, as we have already described, they had lower levels of adrenocortical products (DHEA-S and cortisol). The effect of insulin on adrenal activity is controversial. Some studies suggest that insulin is a negative modulator of adrenal androgen metabolism [37, 38], although some studies using insulin sensitizers in PCOS women showed a decrease of adrenal androgens after their administration [9, 39]; however this event could be consequent to decreases in LH levels that also occurs after metformin administration to these women [39]. We found a significant and negative correlation between basal cortisol and HOMA-IR and fasting insulin levels in PCOS women and a positive correlation between basal cortisol and ISI. Our results are in accordance with Gurusinghe et al. [40] who found that morning cortisol and DHEA-S correlated with ISI in PCOS women. After a linear regression analysis we showed that LH and insulin levels may, respectively, positively and negatively predict cortisol levels. We can thus raise the hypothesis that the lower levels of insulin in nonobese PCOS women might be also contributing to the higher adrenocortical production found in this group compared to obese PCOS women. On the other hand, the higher levels of insulin in the obese PCOS group could also be contributing to a lower androgen production of the adrenal gland.

Also in accordance with our results, Saxena and Seely [28] demonstrated that hyperandrogenic women could be divided into two subgroups: one with insulin resistance (IR), normal, or minimally elevated LH, and markedly elevated insulin levels and one with elevated LH levels, no insulin resistance, and normal insulin concentrations. They found a negative correlation between LH and insulin, but after eliminating the effect of BMI, this correlation was no longer significant. We did not find any correlation between LH and HOMA-IR or ISI suggesting a minor role of LH in PCOS pathophysiology in obese women, when compared to nonobese women.

In conclusion, our results indicate an increased adrenocortical production, not related to insulin resistance, in nonobese PCOS women, and associated with a normal HPA suppression after DEX administration. A neuroendocrine disturbance characterized by higher levels of LH in nonobese PCOS women might be involved in the increased adrenocortical production in this group. Further studies are needed to better clarify the role of adrenal gland in the pathophysiology of PCOS in nonobese women.

## Figures and Tables

**Table 1:** Clinical and biochemical characteristics of all subjects.

	Control	PCOS non obese	PCOS obese
*n*	18	16	21
Presence of amenorrhea	0%	25%*	33%^#^
Age (years)	32.7 ± 6.2	26.1 ± 5.5*	30.4 ± 6.9
BMI (Kg/m^2^)	23.9 ± 3.6	25.2 ± 3.3	37.9 ± 4.7^#§^
Waist (cm)	81.6 ± 8.0	86.1 ± 11.0	114.4 ± 13.8^#§^
Waist-hip ratio	0.9 ± 0.1	0.9 ± 0.1	0.98 ± 0.1^#§^
LH (mIU/mL)	5.6 ± 2.0	13.7 ± 11.3*	6.9 ± 3.6^§^
FSH (mIU/mL)	7.2 ± 1.4	6.1 ± 1.3	5.2 ± 1.2^#^
Androstenedione (ng/mL)	1.7 ± 0.8	3.3 ± 1.9*	2.8 ± 2.1
DHEA (ng/mL)	5.5 ± 3.4	13.8 ± 10.8*	7.8 ± 8.3
DHEA-S (µg/dL)	119.4 ± 61.7	192.9 ± 67.9*	136.6 ± 66.3^§^
Total testosterone (ng/dL)	33.8 ± 12.4	64.8 ± 29.6*	62.0 ± 40.6^#^
SHBG (nmol/L)	59.1 ± 24.8	37.1 ± 20.7*	26.7 ± 13.4^#^
FAI	2.4 ± 1.2	8.5 ± 8.8*	10.0 ± 9.0^#^
Free testosterone (ng/dL)	0.5 ± 0.2	1.3 ± 0.9*	1.4 ± 1.1^#^
Bioavailable testosterone (ng/dL)	11.0 ± 4.3	23.0 ± 11.2	32.0 ± 24.8^#^
Fasting glucose (mg/dL)	90.6 ± 9.4	91.9 ± 11.3	92.7 ± 8.9
Fasting insulin (µU/mL)	6.6 ± 2.7	8.4 ± 5.7	13.5 ± 5.6^#§^
HOMA-IR (µIU/mL)	1.5 ± 0.6	2.0 ± 1.5	3.1 ± 1.4^#§^
Basal cortisol (ng/dL)	1016.6 ± 367.0	1473.0 ± 539.1*	1011.4 ± 512.8^§^
Cortisol after DEX (ng/dL)	198.9 ± 219.7	282.7 ± 329.1	162.7 ± 176.4
∆ cortisol (ng/dL)	817.7 ± 406.7	1190.0 ± 549.7	848.8 ± 489.6
Cortisol supression (%)	−78.7 ± 25.3	−80.8 ± 20.7	−82.5 ± 16.5

*P < .05 PCOS non obese *versus* control.

^#^ *P* < .05 PCOS obese *versus control.*

^§^ *P* < .05 PCOS obese *versus* PCOS non obese.

**Table 2:** Linear regression analysis of the hormomes that influence in cortisol levels among PCOS subjects.

	Beta	95% Confidence Interval	*P*
LH	0.34	3.8–42.0	.020
Fasting insulin	−0.43	−65.9–−13.0	.005
